# Identifying Clinical Characteristics of Young People with Treatment-Resistant Schizophrenia Undergoing Community Initiation of Clozapine

**DOI:** 10.1093/schbul/sbag071

**Published:** 2026-05-09

**Authors:** Oisín Conaty, Andrew Thompson, Patrick McGorry, Fiona Gaughran, Brian O’Donoghue, John Lally

**Affiliations:** Department of Psychiatry, School of Medicine, University College Dublin, Dublin, D04 V1W8, Ireland; Department of Psychiatry, St Vincent’s Hospital Fairview, Dublin, D03 XK40, Ireland; Centre for Youth Mental Health, University of Melbourne, Parkville, Victoria, 3010, Australia; Orygen, Parkville, Victoria, 3052, Australia; Centre for Youth Mental Health, University of Melbourne, Parkville, Victoria, 3010, Australia; Orygen, Parkville, Victoria, 3052, Australia; Department of Psychosis Studies, Institute of Psychiatry, Psychology & Neuroscience, King's College London, London, SE5 8AF, United Kingdom; National Psychosis Service, South London and Maudsley NHS Foundation Trust, London, BR3 3BX, United Kingdom; National Institute for Health Research (NIHR) Mental Health Biomedical Research Centre at South London and Maudsley NHS Foundation Trust and King's College London, London, SE5 8AF, United Kingdom; Department of Psychiatry, School of Medicine, University College Dublin, Dublin, D04 V1W8, Ireland; Centre for Youth Mental Health, University of Melbourne, Parkville, Victoria, 3010, Australia; Orygen, Parkville, Victoria, 3052, Australia; Department of Psychiatry, St Vincent’s University Hospital, Dublin, D04 T6F4, Ireland; Department of Psychiatry, School of Medicine, University College Dublin, Dublin, D04 V1W8, Ireland; Department of Psychiatry, St Vincent’s Hospital Fairview, Dublin, D03 XK40, Ireland; Department of Psychosis Studies, Institute of Psychiatry, Psychology & Neuroscience, King's College London, London, SE5 8AF, United Kingdom

**Keywords:** first-episode psychosis, clozapine, early intervention, antipsychotic, outpatient

## Abstract

**Background and Hypothesis:**

The requirement for hospital admission to initiate clozapine presents a health-systems-related barrier to clozapine prescription and contributes to its underutilization in treatment-resistant schizophrenia (TRS). This study aimed to examine the clinicodemographic characteristics associated with treatment settings for clozapine initiation within a first-episode psychosis (FEP) cohort attending an early intervention in psychosis service.

**Study Design:**

Secondary analysis of a retrospective cohort study of 1220 young people presenting with FEP to the Early Psychosis Prevention and Intervention Centre (EPPIC) in Melbourne between 2011 – 2017.

**Study Results:**

Ninety-one cases of TRS were identified and included in the analysis, with 70 commencing clozapine, of whom 67 had a commencement setting identified. Over half (*n* = 36, 53.7%) commenced clozapine in the community. When compared to the hospital initiation group, the community initiation group were less likely to have had a hospital admission at baseline (odds ratio (OR) 0.26, 95%CI, 0.09-0.87) or an involuntary admission during the 2 year episode of care with EPPIC (OR 0.25, 95%CI, 0.09-0.70). The community initiated group had presented with less severe delusion scores on short form Scale for Assessment of Positive Symptoms at baseline (mean 3.08 vs 3.94, *P* = .031). First generation migrants were less likely to initiate clozapine in the community (OR 0.29, 95%CI, 0.09-0.97). The community initiation group also had reduced odds of clozapine discontinuation until discharge from EPPIC (OR 0.22, 95%CI, 0.06-0.76).

**Conclusion:**

Community initiation provides an alternative route to clozapine treatment and may be associated with a reduced rate of clozapine discontinuation.

## Introduction

Treatment-resistant schizophrenia (TRS) is defined as the persistence of clinically significant symptoms which have not responded to two different trials of antipsychotic medication of adequate dose for at least 6 weeks duration with adequate medication adherence.^1^ Approximately one-third of people with first-episode psychosis (FEP) or schizophrenia (FES) remain symptomatic up to 5 years after illness onset.^2-4^ Among these, up to 70% demonstrate non-response to non-clozapine antipsychotics from illness onset, a pattern described as early treatment-resistance.^3^^,^^4^ Clozapine is the only licensed medication for TRS, and is associated with reduced all-cause mortality,^5^^,^^6^ substance use,^7^^,^^8^ aggression,^9^ and suicide^6^ in this cohort. Despite its established clinical effectiveness, clozapine remains under-prescribed globally.^10^

Multiple patient, clinician, and health system-level barriers contribute to delays in clozapine use. These include the burden of mandatory hematological monitoring; patient reluctance to adhere with blood monitoring; clinician concerns regarding adverse effects and their impact; clinician knowledge of clozapine and/or identification of TRS; and, in many settings, the requirement for inpatient admission to initiate treatment.^11-14^ These barriers contribute to clozapine underutilization and reported delays in initiation of clozapine from illness onset for most patients, ranging from 1 to 14 years.^12^^,^^14^^,^^15^ Such delays are clinically significant, as earlier initiation, particularly within the first 3 years of illness is, associated with improved symptomatic and functional outcomes.^16^^,^^17^ In a qualitative study examining the attitudes of patients toward commencing clozapine, the majority of clozapine eligible patients expressed a preference for initiating clozapine in the community, rather than during hospital admission.^13^ However, community initiation of clozapine is not permitted in some jurisdictions, including Japan, and where inpatient initiation is mandatory, this requirement may represent a substantial structural barrier to timely clozapine use.^18^ Therefore, introducing community clozapine initiation services addresses this health system related barrier to its use in jurisdictions where this is permitted.

Service innovations that facilitate community-based clozapine initiation may improve access. The introduction of the Treatment Review and Assessment Team, a specialist secondary care TRS service in London providing community clozapine initiation increased annual clozapine initiations 5-fold, with more than two-thirds of referrals successfully starting clozapine.^19^^,^^20^ Community initiation pathways may also confer clinical and economic benefits, including reduced hospital bed-days and lower service utilization, with a reduced number of outpatient contacts required over 1-2 years following treatment commencement.^20^ Community initiation services commonly employ standardized assessment to identify clozapine eligible patients.^19^ However, there is a paucity of clinical guidelines for community clozapine initiation, with current guidelines largely limited to exclusion criteria based on medical co-morbidities including myeloproliferative disorders, uncontrolled epilepsy, severe cardiac, or renal disease, previous neuroleptic malignant syndrome or severe paralytic ileus, or an inability to engage with outpatient monitoring as contra-indications.^19^^,^^21^

To date, few studies have examined the clinical pathways leading to community initiation and none to our knowledge have examined demographic and clinical factors associated with community clozapine initiation from diagnosis of FEP.

This study had 2 primary aims: first, to determine the frequency of community- and hospital-based clozapine initiation and to identify demographic and clinical characteristics associated with each setting; and second, to establish the prevalence of TRS and the rate of clozapine use in an FEP cohort.

## Methods

### Setting

The Early Psychosis Prevention and Intervention Centre (EPPIC) is a specialist early intervention in psychosis service within Orygen, Melbourne, Australia. EPPIC offers intervention for young people aged 15-25 years old diagnosed with FEP, for a 2 year episode of care.^22^ EPPIC treats approximately 300 young people diagnosed with FEP per year within a catchment area of approximately one million people.^23^^,^^24^ EPPIC includes separate treatment streams for FEP and At-Risk Mental State (ARMS).^22^ Assessment of treatment-resistance by the Treat Resistance Early Assessment Team (TREAT) and community initiation of clozapine is embedded within this service, supported by structured monitoring and access to inpatient care if required.

In Australia, clozapine is licensed for TRS only from age 16 years^25^; however, in EPPIC, clozapine is prescribed off-license for treatment-resistant schizoaffective disorders and less commonly in non-schizophrenia spectrum psychotic disorders in consultation with the TREAT. The TREAT panel is a multi-professional team who discuss select young people with persistent psychotic symptoms despite treatment and make recommendations for further treatment. The prescription of clozapine in under sixteens is also off-license and considered on a case by case basis in EPPIC.^25^

### Study Design

This study was a secondary analysis, conducted with an exploratory, hypothesis-driven design on an existing retrospective cohort study from EPPIC. Data were previously collected retrospectively from the healthcare records of 1220 young people diagnosed with FEP who attended EPPIC between the years 2011 and 2017**.** As reported by Brown et al., clinical data such as diagnosis at 3 months and discharge, hospital admissions, and antipsychotic prescriptions were collected from electronic patient records, which included initial assessment reports, outpatient notes, clinical review meetings, and discharge summaries. Diagnoses were recorded by the treating consultant psychiatrist.^26^ Only individuals meeting criteria for FEP were included. Individuals diagnosed with ARMS were not included in this analysis. Individuals presenting with persistent hallucinations without functional impairment and without meeting threshold for psychotic disorder were not included in the cohort.

### Participants

There were predefined clinical subgroups within the study. The FEP cohort comprised the full sample of 1220 participants with first-episode psychosis. The following diagnoses were included in the study on the condition that the individual was prescribed clozapine; schizophrenia, schizoaffective disorder, bipolar disorder, delusional disorder, and psychosis not otherwise specified (NOS). Diagnoses were made clinically by the treating consultant psychiatrist following formal assessment at three months and discharge from EPPIC, according to Diagnostic and Statistical Manual of Mental Disorders (DSM) IV Text Revision criteria until 2013, and DSM-5 criteria from 2013 onward.^27^^,^^28^ Diagnosis at discharge was used for the purpose of analysis to record diagnostic evolution over time.

The TRS cohort included individuals meeting criteria for TRS according to the TRRIP consensus definition as outlined in Table 1, as well as those considered clinically eligible for treatment with clozapine. Within the TRS cohort, participants were further divided into TRS-clozapine and TRS non-clozapine groups based on whether clozapine was initiated during the episode of care.

**Table 1:** Operationalization of TRRIP Criteria to Retrospectively Identify TRS in Young People Diagnosed with Schizophrenia Spectrum Disorder but not Commenced on Clozapine

Persistent positive symptoms	The presence of persistent positive symptoms was identified using short form SAPS scores which were rated on a 3-monthly basis during the 2-year episode of care with EPPIC. Data relating to relapses and inpatient admissions were also compared to determine persistent burden of symptoms.
Adequate dose of antipsychotic medication	The maximum dose of each antipsychotic trial was available. A therapeutic dose was achieved if the documented dose for each antipsychotic was ≥600 mg chlorpromazine equivalents (or equivalent therapeutic dose as per Australian Medicines Handbook).^51^
Adequate duration of treatment ≥ 6 weeks	Dates of commencement and discontinuation of each antipsychotic trial were available and compared to ensure at least 6 weeks duration of treatment.
Adequate adherence to antipsychotic medication	The reason for cessation of each antipsychotic trial was available. Trials were not deemed unsuccessful if the reason for discontinuation was recorded as poor adherence. Adequate adherence was assumed for trials of long acting injectable antipsychotics.
Inadequate response to at least two antipsychotic trials	Data relating to each antipsychotic trial was available. An inadequate response was determined if the reason for discontinuation was recorded as poor efficacy, or if short form SAPS scores remained elevated after the commencement date of the trial. Dates of inpatient admission and relapses were also examined to determine if relapse or admission coincided with an unsuccessful antipsychotic trial.

### Identification of Treatment-Resistance

Treatment resistance was defined according to the Treatment Response and Resistance in Psychosis (TRRIP) consensus criteria.^1^ Individuals were classified as having TRS if they had persistent psychotic symptoms despite at least 2 adequate trials of antipsychotic medication of sufficient dose and duration with documented adherence.

Treatment resistance was identified in 2 ways.

First, all individuals commenced on clozapine during their episode of care were considered to have met clinical criteria for clozapine eligibility (TRS-clozapine group).

Second, individuals diagnosed with schizophrenia spectrum disorders who were not prescribed clozapine were examined retrospectively to identify cases meeting TRRIP criteria (TRS-non-clozapine group). Only cases with documented non-response were included; those who discontinued antipsychotics because of intolerance or non-adherence were excluded. The TRRIP criteria, and the data used to determine if young people met criteria are outlined in Table 1.

Within the TRS cohort, participants were categorized as TRS-clozapine or TRS-non-clozapine based on whether clozapine was initiated during the episode of care.

For all young people commenced on clozapine, the treatment setting of clozapine initiation was identified using hospital admission dates and medication data.

### Measures

The severity of positive symptoms was measured using the short form Scale for the Assessment of Positive Symptoms (SAPS)^29^ on entry to the service (baseline), and every 12 weeks while in the service. The level of functioning was assessed using the Global Assessment of Functioning (GAF)^27^ and Health of Nation Outcome Scale (HoNOS)^30^ scales at baseline and at discharge, at the end of the 2 year episode of care.

Time to clozapine initiation from first antipsychotic prescription was calculated from the date of first prescription of antipsychotic medication to commencement of clozapine. Duration of untreated psychosis (DUP) was determined retrospectively by clinicians in EPPIC on first assessment and calculated from the first presentation of psychotic symptoms until the commencement of antipsychotic medication. Time to clozapine initiation from illness onset was calculated by combining the DUP and time to clozapine initiation from first antipsychotic prescription.

Migrant status and ethnicity data were recorded separately from the retrospective chart review. First generation migrant status was recorded, and subsequent generations were counted as being born in Australia. Ethnicity data was based on self-identification.

Hospital admissions and legal status, relapses, medication use, maximum clozapine dose until leaving care in EPPIC, substance use, and clozapine discontinuation within the episode of care in EPPIC were also recorded.

Admission status during episode of care was defined as an admission which occurred after first presentation to EPPIC and during the two year episode of care. Admissions during the episode of care were coded into voluntary and involuntary admissions. A relapse was determined by the treating consultant psychiatrist. Substance use during episode of care was defined as substance use meeting criteria of at least harmful use or mild substance use disorder or more severe as rated by clinicians in EPPIC according to DSM criteria during the two year episode of care.^27^^,^^28^

### Outcomes

The co-primary outcomes were the proportion of community versus hospital clozapine initiation; and the prevalence of TRS and the rate of clozapine use among individuals meeting criteria for TRS.

Secondary outcomes included clinical and demographic predictors of initiation setting, time to clozapine, substance use, admissions, and clozapine discontinuation.

### Statistical Analysis

Descriptive statistics of the baseline characteristics were carried out for the study cohort. Data were examined to determine if parametric or non-parametric. Tests of skewness were applied. Subgroups of interest were created (TRS-clozapine vs TRS-non-clozapine; community vs hospital clozapine initiation) and differences in baseline characteristics were compared using independent sample *t*-tests (parametric data) or Mann–Whitney *U* tests (non-parametric data). Categorical variables were analyzed using χ^2^ tests, and odds ratios (ORs) with 95% CIs were calculated.

Logistic regression models were used to examine factors associated with community versus hospital initiation; and TRS-clozapine versus TRS-non clozapine. Logistic regression was used to examine associations between independent variables; (age, gender, first generation migrant status, baseline hospitalization, admission status during episode of care, substance use during episode of care, delusion item score at baseline on short form SAPS, and time to clozapine initiation from first antipsychotic prescription) and the setting of clozapine initiation (ie, community vs hospital initiation), and similarly for the TRS-clozapine vs TRS-non-clozapine comparison. For the purpose of this exploratory study, independent variables were selected based on clinical relevance and univariate associations (*P* < .25).

In the regression analyses, the reference category for the comparison of TRS-clozapine versus TRS-non-clozapine was the TRS-non-clozapine group, and hospital initiation was used as the reference category for analyses examining setting of treatment initiation. Logistic regression was performed using a backward stepwise likelihood ratio method. Variables with more than 40% missing data were excluded from the models: these included baseline GAF score and DUP.

As this was an exploratory study, no adjustment for multiple comparisons was performed.^31^^,^^32^ Given the hypothesis-driven exploratory design, the aim was to minimize the risk of Type II error (false negative findings), which may increase when strict correction for multiple testing is applied, although this approach may increase the likelihood of Type I error. In addition, clinical variables are often inter-correlated, and therefore the use of the Bonferroni correction may not be appropriate, as it assumes independence between statistical tests.^33^

Analysis was performed using SPSS (Version 29.0, IBM SPSS Statistics, USA).

### Ethical Approval

This study was approved as a quality assurance project by the Melbourne Health Research Ethics Committee (QA2024155). The authors confirm that all procedures contributing to this study comply with the Declaration of Helsinki 1945, as revised in 2008. Individual consent was deemed not to be required for this retrospective cohort analysis.

## Results

**Figure 1:**
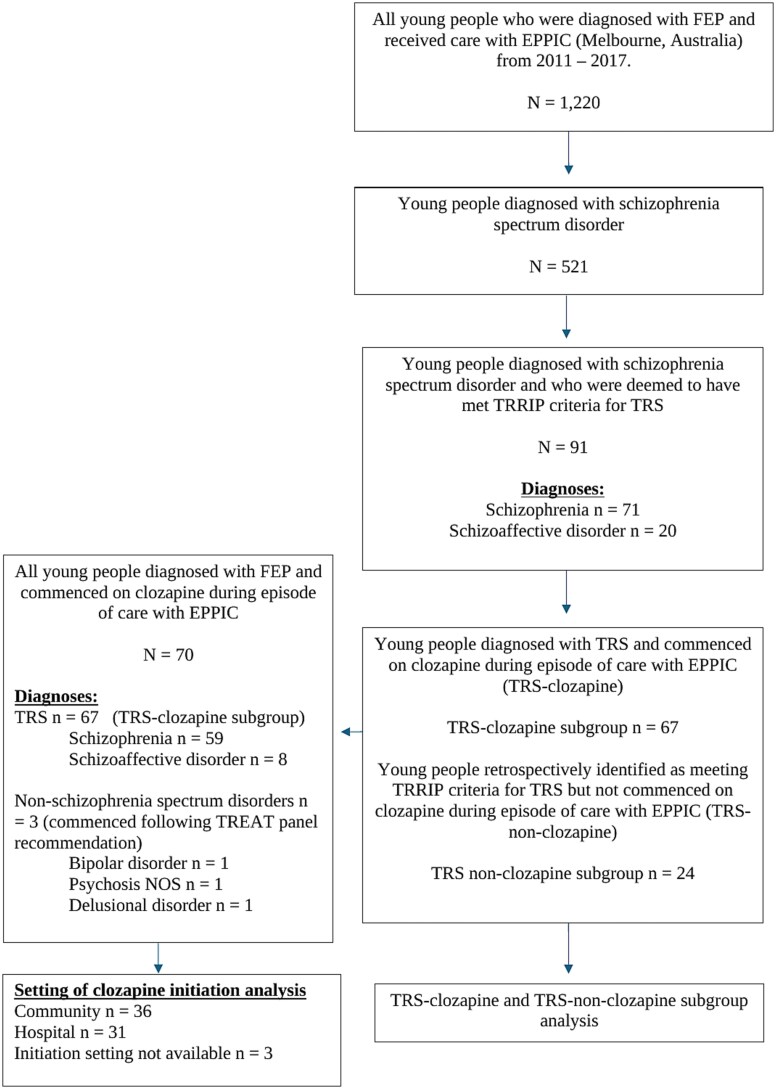
Selection of Study Cohort and Sub-group.

Figure 1 outlines the primary cohort and sub-group identified for analysis. Of the 1220 young people who received care in EPPIC in the timeframe of interest, 91 were identified as having TRS, and a further 3 young people diagnosed with non-schizophrenia spectrum disorders were deemed to fulfill clozapine eligibility by the TREAT panel (*n* = 3). Seventy patients were commenced on clozapine during their episode of care with EPPIC, of whom 96.7% (*n* = 67) had a diagnosis of TRS/schizoaffective disorder. Initiation setting was available for 67 people starting clozapine. Eleven young people who registered with EPPIC at the age of fifteen for treatment of FEP commenced clozapine during their 2 year episode of care (*n* = 11). The exact age at clozapine initiation was not available. However, within these 11 individuals, the mean time to clozapine initiation from first prescription of antipsychotic medication was 66.6 weeks (SD ± 40.3).

### Clinical and Demographic Characteristics

Ninety-four young people who received care in EPPIC were identified as meeting criteria for clozapine use and included. The demographic and clinical characteristics of this cohort are outlined in Table 2. Diagnoses in the clozapine eligible group were schizophrenia (75.5%, *n* = 71), schizoaffective disorder (21.2%, *n* = 20), bipolar disorder with psychotic symptoms (1.1%, *n* = 1), psychosis NOS (1.1%, *n* = 1), and delusional disorder (1.1%, *n* = 1). One young person diagnosed with bipolar disorder had been commenced on clozapine. However, other cases of bipolar disorder were not included, nor examined for treatment resistance.

**Table 2:** Clinical and Demographic Characteristics of Clozapine Eligible Cohort

	***N* (*N* = 94)**	**%**
**Sex**		
Male	55	58.5
Female	39	41.5
**Ethnicity**		
Caucasian	13	13.8
Asian	12	12.8
African	9	9.6
Middle Eastern	3	3.2
Aboriginal	1	1.1
Māori	2	2.1
Polynesian	1	1.1
Not available	53	56.4
**Migrant status**		
Born in Australia	75	79.8
First generation migrant	19	20.2
**Diagnosis**		
Schizophrenia	71	75.5
Schizoaffective disorder	20	21.3
Bipolar disorder with psychotic features	1	1.1
Delusional disorder	1	1.1
Psychosis not otherwise specified	1	1.1
	**Median**	**IQR**
Age (years)	19	17-21
Duration of untreated psychosis (weeks)	12	3-52
No. antipsychotic trials	3	2-4
Time to clozapine initiation from first antipsychotic prescription (weeks)	62	38-85
Time to clozapine initiation from illness onset (weeks)	81	50-118
Max clozapine dose (mg)	325	250-450
	**Mean**	**SD**
**Symptom Measures**		
SAPS baseline	9.0	4.2
SAPS week 108 (discharge)	2.5	2.9
**Functioning Measures**		
GAF baseline	46.9	13.2
GAF discharge	57.8	14.7
HoNOS baseline	16.5	8.0
HoNOS discharge	11.3	7.6

Abbreviations: GAF = Global Assessment of Functioning^27^; HoNOS = Health of Nation Outcome Score^30^; SAPS = Scale of Assessment of Positive Symptoms.^29^

First generation migrant status was available for all young people (*n* = 94), with 20.2% of young people being first generation migrants. Self-identified ethnicity was available for 43.6% (*n* = 41) and outlined in Table 2. The limited ethnicity data available should be interpreted cautiously.

Seventy (74.5%) of those who were clozapine eligible were commenced on clozapine during their episode of care in EPPIC. Of those starting clozapine, 30% (*n* = 21) had received at least one trial of long-acting injectable antipsychotic and 19.1% (*n* = 18) had received antipsychotic polypharmacy after meeting TRS criteria and prior to commencing clozapine.

Symptom and functioning measures are outlined in Table 2.

### TRS-Clozapine and TRS-Non-clozapine Groups

Differences between the young people diagnosed with TRS who commenced clozapine (TRS-clozapine) (*n* = 67) and those who did not (TRS-non-clozapine) (*n* = 24) were examined. Compared with the TRS-non-clozapine group, the TRS-clozapine group had an increased likelihood of being male (OR 3.83, 95% CI, 1.43-10.27) and had increased odds of being admitted during their episode of care with EPPIC (OR 5.53, 95% CI, 1.21-25.28). The TRS-clozapine subgroup had fewer antipsychotic trials (mean 3, SD ± 1.13) than the TRS-non-clozapine subgroup (mean 3.95, SD ± 1.1) [mean difference 0.95, *P* = .001].

The timing of substance use in relation to attendance in EPPIC was examined between the TRS-clozapine and TRS-non-clozapine groups. There was reduced odds of substance use at the time of presentation (OR 0.22, 95% CI, 0.08-0.59); and during the episode of care in EPPIC (OR 0.36, 95% CI, 0.14-0.94) in the TRS-clozapine group.

In multivariate analysis, male sex (adjusted OR 7.1, 95% CI, 1.7-30.9), hospital admission of any legal status during episode of care (adjusted OR 4.4, 95% CI, 1.0-19.5) and reduced likelihood of substance during the episode of care (adjusted OR 0.15, 95% CI, 0.04-0.60), were significantly associated with clozapine initiation in the TRS-clozapine subgroup.

### Setting of Clozapine Initiation

Over half the young people completed their clozapine initiation titration in the community (*n* = 36, 53.7%). Over 96% of the cohort in which commencement setting could be identified were diagnosed with TRS, however, this cohort also included non-schizophrenia spectrum disorders (*n* = 3). Table 3 outlines the differences between the community and hospital initiation groups.

**Table 3:** Setting of Clozapine Initiation

	**Total (*n* = 67)** ^**a**^	**Community (*n* = 36)**	**Hospital (*n* = 31)**	**Univariate analysis**		**Multivariate analysis**	
**Sex**	***n* (%)**	***n* (%)**	***n* (%)**	**OR (95% CI)**	***P***	**aOR (95% CI)**	***P***
Male	47 (70.2)	23 (63.9)	24 (77.4)				
Female	20 (28.9)	13 (36.1)	7 (22.6)	1.9 (0.7-5.7)	.23	–	–
**Diagnosis**	***n* (%)**	***n* (%)**	***n* (%)**				
Schizophrenia	56 (83.6)	30 (83.3)	26 (83.9)				
Schizoaffective disorder	8 (11.9)	4 (11.1)	4 (12.9)				
Bipolar disorder with psychotic features	1 (1.5)	0 (0)	1 (3.3)				
Delusional disorder	1 (1.5)	1 (2.8)	0 (0)				
Psychosis not otherwise specified	1 (1.5)	1 (2.8)	0 (0)				
**Migrant status**	***n* (%)**	***n* (%)**	***n* (%)**	**OR (95% CI)**	***P***	**aOR (95% CI)**	***P***
First generation migrant	16 (23.9)	5 (13.9)	11 (35.5)	0.3 (0.1-1.0)	.04	0.02 (0-0.2)	.02
Born in Australia	51 (76.1)	31 (86.1)	20 (64.5)				
**Hospital admissions**	***n* (%)**	***n* (%)**	***n* (%)**	**OR (95% CI)**	***P***	**aOR (95% CI)**	***P***
Admission of any legal status at baseline	44 (65.7)	19 (52.8)	25 (80.7)	0.3 (0.1-0.9)	.02	0.2 (0.1-0.8)	.03
Involuntary admission at baseline	25 (37.3)	9 (25.0)	16 (51.6)	0.3 (0.1-0.9)	.03	0.2 (0.02-1.4)	.1
Admission after presentation	58 (86.6)	28 (77.8)	30 (96.8)	0.16 (0.02-1.37)	.06	–	–
Involuntary admission after presentation	38 (56.7)	15 (41.7)	23 (74.2)	0.25 (0.09-0.70)	.01	0.1 (0.1-0.7)	.02
**Clinical factors**		**Mean (SD)**	**Mean (SD)**	**t, df / U**	***P***		
Duration of untreated psychosis (weeks)		50.0 (85.5)	28.6 (34.3)	1.2, 56	.24		
No. relapses		1.1 (1.6)	1.2 (1.4)	−0.3, 64	.78		
Total no. admissions		2.4 (2.9)	4.3 (2.6)	−2.7, 65	.01		
No. admissions prior to clozapine initiation^b^		1.8 (1.3)	2.1 (1.3)	−0.9, 56	.38		
No. admissions after clozapine initiation		0.3 (0.97)	0.7 (1.9)	−1.0, 56	.32		
No. antipsychotic trials prior to clozapine		2.8 (1.1)	3.3 (1.1)	696.5	.07		
Time to clozapine initiation from illness onset (weeks)		123.9 (108.6)	74. (43.3)	234	.08		
Duration of follow-up after clozapine initiation (weeks)		43.1 (28.9)	51.1 (34.6)	−0.9, 57	.36		
**Symptom Measure (SAPS) at baseline**		**Mean (SD)**	**Mean (SD)**	**t, df**	***P***		
Hallucinations		2.9 (1.8)	2.7 (1.7)	0.4, 65	.67		
Delusions		3.1 (1.9)	3.9 (1.2)	−2.2, 65	.03		
Bizarre behavior		1.6 (1.7)	1.5 (1.8)	0.1, 64	.96		
Formal thought disorder		1.5 (1.7)	1.2 (1.5)	0.6, 64	.55		
Total score		8.9 (4.8)	9.5 (3.5)	−0.52, 64	.61		
**Functional Measures at baseline**		**Mean (SD)**	**Mean (SD)**	**t, df**	***P***		
GAF baseline		17.0 (27.6)	12.1 (1.6)	0.7, 53	.48		
HoNOS baseline		14.2 (7.2)	18.1 (8.3)	−1.7, 45	.09		

aOR = adjusted odds ratio; GAF = Global Assessment of Functioning^27^; HoNOS = Health of Nation Outcome Score^30^; OR = odds ratio; SAPS = Scale of Assessment of Positive Symptoms^29^.

^a^The setting of clozapine initiation could not be identified in 3 cases and therefore excluded from the analysis by initiation setting.

^b^The admission during which clozapine was initiated is excluded from the total of admissions prior to clozapine initiation.

Among symptom measures, the only significant difference between groups was on the delusion item subscale of the short form SAPS measured at baseline, with lower scores in the community initiation group, with a mean of 3.08 (SD ± 1.87) compared to the mean of 3.94 (SD ± 1.15) in the hospital initiation group (*t* = −2.20, *P* = .03). There were no other significant symptom differences at baseline or at other time points. The community initiation group also had reduced odds of substance use during their episode of care (OR 0.36, 95% CI, 0.13-0.99).

Time from first antipsychotic to clozapine was significantly longer in the community group than in the hospital initiation group (mean 69.2 (SD ± 45.20) vs 48.5 weeks (SD ± 33.25), *t* = 2.10*, P* = .039).

Fewer additional antipsychotic trials after the second adequate trial were observed in the community group compared to the hospital group (mean 0.45 (SD ± 0.77) vs 1.23 (SD ± 1.21) (U = 559.5, *P* = .006)).

Being a first-generation migrant (OR 0.29, 95% CI, 0.09-0.97), having a hospital admission of any legal status at baseline (OR 0.3, 95% CI, 0.1-0.9), and having an involuntary admission after first presentation (OR 0.25, 95% CI, 0.09-0.70) were associated with a reduced likelihood of clozapine community initiation. In multivariate analysis, community clozapine initiation remained associated with reduced likelihood of being a first generation migrant (adjusted OR 0.02, 95% CI, 0.01-0.24), reduced likelihood of hospital admission of any legal status at baseline (adjusted OR 0.06, 95% CI, 0.01-0.63), and reduced likelihood of involuntary admission after presentation (adjusted OR 0.14, 95% CI, 0.03-0.72).

Sensitivity analysis restricted to schizophrenia spectrum disorder produced similar results. In multivariate analysis of the TRS-clozapine subgroup only, reduced likelihood of being a first generation migrant (adjusted OR 0.05, 95% CI, 0.01-0.38) and reduced likelihood of hospital admission of any legal status at baseline (adjusted OR 0.19, 95% CI, 0.04-0.9) remained statistically significant.

### Clozapine Discontinuation

Sixteen individuals (22.86%; 12 male and 4 female), discontinued clozapine during follow up. Those in the community initiation group had a reduced likelihood of discontinuation (OR 0.22, 95% CI, 0.06-0.76). Those in the community initiation group had a mean follow-up post-clozapine commencement of 43.14 weeks (SD ± 28.93), with a mean follow up of 51.08 weeks (SD ± 34.58) in the hospital initiation group. Clozapine discontinuers had significantly lower mean maximum clozapine dose (232.69 mg (SD ± 96.49)), compared to those who remained on clozapine treatment (mean dose of 383.67 mg (SD ± 147.86) (*t* = −3.48, *p* ≤ .001)). Clozapine discontinuation reasons are outlined in Table 4. Data on reasons for discontinuation were missing for 4 of the 16 cases. Of the 16 cases, 7 young people (46.7%) discontinued clozapine within 6 weeks of commencement; 5 of whom initiated clozapine in hospital, 1 in the community, with the setting of initiation unavailable for 1 case. Adjusted analysis for discontinuation was not performed due to limited events.

**Table 4:** Clozapine Discontinuation Analysis

	**Total**	**Community initiation**	**Hospital initiation**	**Statistical test of difference**	**Significance**
	***n***	***n* (%)**	***n* (%)**	**OR (95% CI)**	***P***
No. young people who discontinued clozapine^a^	16	4 (25)	11 (68.75)	0.2 (0.1-0.8)	.02
**Sex**					
Male	12	3 (33.3)	9 (66.6)	0.7 (0.2-2.5)	.56
Female^a^	4	1 (25)	2 (50)		
					
Discontinued clozapine within 6 weeks^a,b^	7	1 (14.2)	5 (71.4)	0.6 (0-8.7)	.71
Discontinued clozapine after 6 weeks^b^	8	2 (25)	6 (75)		
		**Mean (SD)**	**Mean (SD)**	**t, df**	***P***
Time to clozapine discontinuation (weeks)		17 (17.1)	10.6 (11.1)	0.8, 12	.44
**Reasons for discontinuation** ^**a**^	***n* (%)**	***n* (%)**	***n* (%)**		
Adverse event	1 (6.3)	0	1		
Non-adherence	4 (25)	2	2		
Not effective	3 (18.8)	0	2		
Other—not listed	4 (25)	0	4		
Missing data	4 (25)	2	2		

Abbreviation: OR = odds ratio.

^a^One case of clozapine discontinuation cannot be grouped based on initiation setting.

^b^Time of clozapine discontinuation cannot be determined in one case.

## Discussion

To our knowledge, this is the first cohort study in FEP to examine clinicodemographic variables associated with the setting of clozapine initiation. We identified that 75% of those with TRS commenced clozapine and that 54% of those treated with clozapine were initiated in the community. Community initiation was associated with reduced rates of clozapine discontinuation in this group compared with hospital initiation.

While this cohort consists of young people initially presenting with FEP, it is best characterized as an early psychosis cohort, as it included those who relapsed during their episode of care. In this study, the prevalence of clozapine eligibility was 7.7% of the total FEP cohort. It was previously demonstrated that less than 10% of young people attending EPPIC met criteria for TRS during their episode of care.^34^ In the present study, 75% of those with TRS commenced clozapine in contrast to global prescription rates among clozapine eligible patients, which range from 25% in Queensland, Australia,^35^ 26% in Spain,^36^ and up to 50% in the United Kingdom,^37^ although these study populations were not limited to the first two years from first presentation.

A retrospective cohort study from three early intervention services in Canada reported that 55% of patients prescribed clozapine were initiated in the community, among 146 individuals with FEP, of whom 29 received clozapine.^38^ This is comparable to the 54% rate of community initiation observed in the EPPIC cohort. The present study extends these findings by examining clinical characteristics associated with initiation setting and discontinuation rates based on the clozapine initiation setting.

There are several service components which may influence the high clozapine uptake in EPPIC. The existence of a multi-disciplinary panel (TREAT panel) focused on identifying treatment-resistance and clozapine eligibility promotes the earlier use of clozapine within the patient population. Second, the accessibility of the community initiation pathway facilitates clozapine uptake. Furthermore, a specialist clozapine monitoring clinic was introduced in EPPIC in 2016 which may also have contributed to improved uptake by streamlining hematological monitoring and providing additional clinical support. This clinic was introduced in addition to existing services, including the TREAT panel. In a pre- and post-evaluation study, the time from first presentation to clozapine initiation post-introduction of the clinic was reduced from 72 to 53.5 weeks among the cohort who attended the clinic. The discontinuation rate of clozapine also reduced, with 14.7% of those who attended the clinic discontinuing clozapine post-introduction, compared to 43.5% prior.^34^ This multi-component service model is benefitted by the specialist nature of the EPPIC service and may not be generalizable to less resourced countries or services. In addition to the multidisciplinary TRS review panel, structured monitoring framework and dedicated clozapine clinic with multidisciplinary support; accessible pathology services for adequate hematological monitoring, and capacity for rapid hospital admission in case of complications arising are considered by the researchers to be essential components of a comprehensive community clozapine initiation service.

Promoting the early use of clozapine when indicated is a core feature of clinical guidelines for the treatment of FEP and early psychosis globally.^39-41^ Early intervention services aim to improve functioning and prevent long term disability. There is a natural link and transition to rehabilitation and assertive outreach services for clozapine-treated patients who have achieved an incomplete functional recovery or whom have difficulties engaging with aspects of treatment, for example, hematological monitoring.^41^^,^^42^

Community initiation removes the barrier of hospital admission in order to access clozapine, the gold standard intervention for TRS, facilitating a five-fold increase in titrations annually in one service.^19^^,^^20^ Studies relating to community clozapine initiation are very limited, with the literature originating in the South London and Maudsley NHS Foundation Trust, UK. The South London population differs significantly from our study population, based on age, with a mean age of 39 years old, and differences in ethnicity.^20^

Differences between community and hospital initiation groups appeared largely related to illness severity and service use. Few differences in baseline symptom profile were identified between the groups, although those in the community initiation group had lower baseline delusion scores on SAPS. Individuals started on clozapine in hospital were more likely to have been admitted at presentation, to have experienced involuntary admission, and to have higher baseline delusion severity, indicating greater clinical acuity. These findings suggest that treatment setting decisions were influenced primarily by risk and symptom severity rather than demographic factors alone. Residual confounding by illness severity is therefore likely, particularly as measures of agitation, behavioral disturbance, and suicide risk were not available in the dataset.

Another notable finding in this study is the longer time to clozapine initiation from first antipsychotic prescription of 69 weeks in the community group, compared to 48 weeks among those who commenced clozapine in hospital*.* This may reflect difference in clinical trajectory with lower rates of hospital admission in the community initiation group both at presentation and during follow up. Furthermore, individuals requiring admission may experience more persistent or severe symptoms leading to earlier escalation of treatment and clozapine use during inpatient care. Alternatively, patients managed primarily in the community may have had fewer opportunities for rapid medication changes, resulting in longer delays before clozapine was considered. Nevertheless, the overall time to clozapine initiation in this cohort (median 62 weeks) was shorter than reported in many previous studies, in which delays of several years are common (23), supporting the effectiveness of structured early intervention pathways in reducing treatment delay. This is consistent with prior evidence that individuals treated in EPICC had shorter delays to commencing clozapine when eligible.^24^ However, previous studies have not examined the setting for clozapine initiation.

The all-cause clozapine discontinuation rate of 22.9% during the episode of care in this cohort is lower than the rates reported in previous studies, which range from 26.8% to 57.0%,^43-47^ although it is higher than the 13.2% discontinuation rate reported in a single Canadian study.^48^ Discontinuation was less frequent in those who initiated clozapine in the community. Using clozapine discontinuation within 6 weeks of commencement as a proxy marker for titration-related intolerability did not demonstrate a significant difference between groups, although this analysis was limited by the small number of discontinuations observed (*n* = 16). To our knowledge, no other study has previously examined the association between the setting of clozapine initiation and subsequent discontinuation.

Although causal inference cannot be made, this finding may reflect differences in titration practice, patient selection, or engagement with treatment. Community initiation protocols typically recommend slower dose escalation than inpatient titration (19, 21), which may reduce early adverse effects such as hypotension, tachycardia, sedation, and myocarditis. Slower titration may allow greater physiological adaptation and improve tolerability (48-50), potentially contributing to improved continuation rates. However, information on titration schedules and plasma concentrations was not available, and reasons for discontinuation were incompletely recorded, limiting interpretation.

### Clinical Implications

Several clinically relevant findings emerge from this study. More than half of patients commenced clozapine in the community, and this model of care may have contributed to the high proportion of eligible patients who commenced clozapine. Community initiation appears to improve accessibility, particularly for patients who were less likely to require a hospital admission during their episode of care and who may have been reluctant to agree to admission for clozapine titration if community initiation was not an option. Careful patient selection appears important, and community initiation may be most appropriate for patients with less severe delusions, lower rates of substance use, and fewer involuntary admissions. In contrast, hospital initiation may be more appropriate for patients who have required more admissions, particularly with involuntary admission status. Patients presenting with higher clinical acuity and risk profile, including severe behavioral disturbance are also likely most appropriate for hospital initiation, although these factors were not assessed in this study. This study highlights the benefit of specialist service structures, including multidisciplinary review, structured monitoring, and dedicated clozapine clinics, which may be key components in achieving higher rates of appropriate clozapine use in early psychosis services.

There are currently no international consensus guidelines for community clozapine initiation, and existing guidelines are largely service-specific, such as those used in the South London and Maudsley NHS Foundation Trust, UK.^21^ Community clozapine initiation is resource-intensive due to the level of monitoring required, access to laboratory testing, and the capacity for rapid admission if complications arise, and typically involves coordinated input from outpatient clinics, day hospitals, or home based treatment teams. However, despite the resources required, our findings suggest that community initiation may be associated with lower clozapine discontinuation rates. Economic evaluations have demonstrated cost savings with community initiation compared to hospital initiation, with reductions in the cost of clozapine initiation and subsequent service utilization, supporting the feasibility, and value of developing community-based initiation pathways. Community initiation costs were reduced by 85% when compared to a 2 week inpatient admission for initiation in a Western Australian service.^49^ Further, community clozapine initiation reduces hospitalization and contact with outpatient mental health services over a two year period post-initiation, with a median saving of over £1600 (AUS$3200/€1900),^20^^,^^50^ thus justifying costs in providing a community clozapine initiation service.

### Strengths and Limitations

This study was conducted in a large, naturalistic cohort of young people with FEP, including all cases attending EPPIC without exclusion criteria during the time period, allowing examination of a broad range of clinical and demographic variables among those who became clozapine eligible. While the sample size initiated on clozapine is modest, this is the first study in FEP to examine factors associated with community based clozapine initiation.

Several limitations should be considered. First, data were collected retrospectively from clinical records and identification of treatment-resistant cases in those not commenced on clozapine may have been incomplete. Second, the clozapine treated subgroup sample size provided limited power for multivariable analyses, particularly for discontinuation outcomes. The relatively young age of the cohort, and incomplete ethnicity data, which was documented for 44% (*n* = 41) of young people, may limit generalizability. Information on smoking status, which can influence clozapine concentrations and dosing, was not available. In addition, potentially important determinants of treatment setting, such as patient preference, family involvement, medical comorbidity, and social support, were not recorded. However, given the young median age of the cohort, medical co-morbidities are expected to be less likely and therefore community initiation more suitable in this population. Finally, as this was an exploratory study, no adjustment for multiple comparisons was performed and consequently, the findings should be considered preliminary pending replication in larger prospective cohort studies.

### Future Directions

Prospective cohort studies are needed to compare community and hospital clozapine initiation in individuals with TRS, to further establish clinical and demographic factors which influence the decision with a view to predicting those who will be successfully titrated in each setting. Specifically, we recommend systematic recording of clozapine titration rates, adverse events and patient, and family preferences. Further work should examine reasons for clozapine discontinuation following hospital and community initiations, particularly if there is a relationship with clozapine titration rates. A mixed-methods study design with a qualitative study to further examine reasons for increased clozapine uptake and lower discontinuation rates observed with community initiation in this population, including patient, family, or carer, and clinician views on the community initiation experience would also be of benefit. In addition, pragmatic service-level evaluations of community-based clozapine initiation programs are recommended, focusing on the quality, safety, effectiveness, and cost of the service as delivered in routine clinical practice to patients starting clozapine.

## Conclusion

In this FEP cohort, three quarters of young people attending EPPIC who met criteria for TRS commenced clozapine, with the majority doing so in the community. Patients with community initiation of clozapine had lower baseline delusion severity, fewer involuntary admissions, and lower rates of substance use, suggesting lower overall clinical acuity. Importantly, community clozapine initiation was associated with a reduced likelihood of clozapine discontinuation during follow up, supporting the feasibility and potential advantages of structured community initiation pathways.

These findings suggest that the availability of a dedicated treatment-resistance review process, specialist clozapine monitoring, and a supported community initiation pathway may contribute to higher clozapine uptake in FEP services. Removing the requirement for hospital admission may reduce system-level barriers to timely clozapine use, particularly for patients who would otherwise be reluctant or unlikely to agree to inpatient treatment.

Although exploratory and based on retrospective data, this study provides the first evidence in a FEP cohort describing clinical factors associated with the setting of clozapine initiation. Prospective studies are required to confirm these findings, to define optimal criteria for community initiation, and to determine whether outpatient titration improves long-term adherence, safety, and clinical outcomes. Development of consensus guidelines for community clozapine initiation may facilitate wider implementation of this model of care and improve access to the most effective treatment for TRS.
